# RELEVANT YET UNCONTROLLABLE: PERCEIVED CONTROL AS A MEDIATOR OF CROSS-CULTURAL DIFFERENCES IN OLD-AGE PREPARATION

**DOI:** 10.1093/geroni/igac059.507

**Published:** 2022-12-20

**Authors:** Helene Fung, Nicole Long Ki Fung, Dwight Cheuk Kit Tse

**Affiliations:** The Chinese University of Hong Kong, Hong Kong, Hong Kong; The Chinese University of Hong Kong, Hong Kong, Hong Kong; University of Strathclyde, Glasgow, Scotland, United Kingdom

## Abstract

Previous studies have shown that there are cross-cultural differences in old-age preparation rate (e.g. Kornadt et al., 2019). Drawing from the transactional stress-and-coping model (Lazarus & Folkman, 1984), we proposed that perceived control, self-relevance and responsibility for old-age preparation could mediate the cultural differences in old-age preparation. We recruited a sample aged 18 to 96 from Germany (N=366, Mage=52.63) and Hong Kong (N=252, Mage=57.47) to complete two online questionnaires across two years. Compared with German adults, Hong Kong adults prepared less (b=-2.159, p<.001), had lower perceived control (b=-0.899, p<.001) and responsibility (b=-0.713, p<.001), yet similar level of self-relevance over preparation. Preparation at time2 was related to self-relevance (b=1.004, p<.001) and control (b=0.785, p<.001) at time1. The cultural differences in preparation at time2 were only mediated by perceived control at time1 (indirect effect=0.706, p<.001). Findings highlight the importance to enhance individual perceived control over old age in promoting society-wide old-age preparation.

